# 1-(4-Chloro­phen­yl)-1*H*-pyrazol-3-ol

**DOI:** 10.1107/S1600536809053641

**Published:** 2009-12-19

**Authors:** Xiao-Yan Ren, Jian-Gang Wang, Yun-Ying Li

**Affiliations:** a Chemical Engineering Department, Weifang Vocational College, Weifang 261000, People’s Republic of China; b Bioengineering School, Weifang University, Weifang 261061, People’s Republic of China; c The 7th Middle School, Weifang 261000, People’s Republic of China

## Abstract

In the title compound, C_9_H_7_ClN_2_O, the dihedral angle between the aromatic ring planes is 11.0 (2)°. In the crystal, inversion dimers linked by pairs of O—H⋯N hydrogen bonds generate *R*_2_^2^(8) loops.

## Related literature

For a related structure, see: Jian *et al.* (2005 ▶). For background to herbicides and plant-growth promoters related to the title compound, see: Shi *et al.* (1995 ▶); Xu *et al.* (2002 ▶).
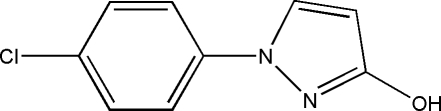


## Experimental

### 

#### Crystal data

C_9_H_7_ClN_2_O
*M* *_r_* = 194.62
Monoclinic, 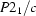

*a* = 9.6461 (19) Å
*b* = 13.833 (3) Å
*c* = 6.5045 (13) Å
β = 94.33 (3)°
*V* = 865.4 (3) Å^3^
*Z* = 4
Mo *K*α radiation
μ = 0.40 mm^−1^
*T* = 293 K
0.11 × 0.09 × 0.08 mm

#### Data collection

Bruker SMART CCD diffractometer
5771 measured reflections
1357 independent reflections
1171 reflections with *I* > 2σ(*I*)
*R*_int_ = 0.038

#### Refinement

*R*[*F* ^2^ > 2σ(*F* ^2^)] = 0.059
*wR*(*F* ^2^) = 0.146
*S* = 1.24
1357 reflections
122 parameters
H atoms treated by a mixture of independent and constrained refinement
Δρ_max_ = 0.23 e Å^−3^
Δρ_min_ = −0.32 e Å^−3^

### 

Data collection: *SMART* (Bruker, 2001 ▶); cell refinement: *SAINT* (Bruker, 2001 ▶); data reduction: *SAINT*; program(s) used to solve structure: *SHELXS97* (Sheldrick, 2008 ▶); program(s) used to refine structure: *SHELXL97* (Sheldrick, 2008 ▶); molecular graphics: *SHELXTL* (Sheldrick, 2008 ▶); software used to prepare material for publication: *SHELXTL* .

## Supplementary Material

Crystal structure: contains datablocks global, I. DOI: 10.1107/S1600536809053641/hb5279sup1.cif

Structure factors: contains datablocks I. DOI: 10.1107/S1600536809053641/hb5279Isup2.hkl

Additional supplementary materials:  crystallographic information; 3D view; checkCIF report

## Figures and Tables

**Table 1:** Hydrogen-bond geometry (Å, °)

*D*—H⋯*A*	*D*—H	H⋯*A*	*D*⋯*A*	*D*—H⋯*A*
O1—H1⋯N2^i^	0.86 (4)	1.89 (4)	2.744 (4)	173 (4)

Symmetry code: (i) 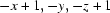.

**Fig. 1.**
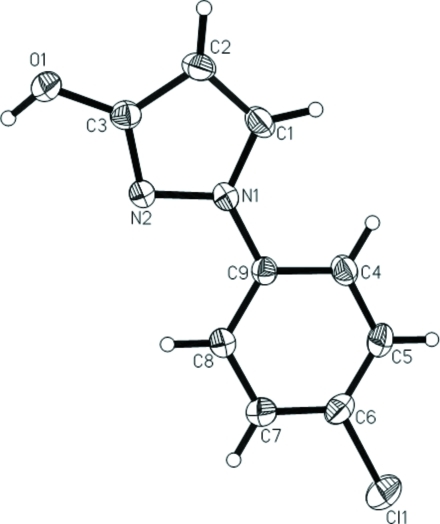
The molecular structure of (I) with displacement ellipsoids drawn at the 30% probability level.

## supplementary crystallographic information

### Comment

*p*-Chlorophenyl hydrazine hydrochloride is an important biologically active compound used in herbicides and plant growth substances (Shi *et al.*,1995; Xu, *et al.*,2002). Here we report the crystal structure of the title compound (I).

In the title compound (I) (Fig. 1), the The dihedral angle between the pheny ring (C4,C5,C6,C7,C8 and C9) and ring 1(N1,N2,C1,C2 and C3) is 11.0 (2)°. The C—N bonds length in the range of (1.321 (5) Å-1.416 (5) Å) are in agreement with that observed before (Jian *et al.*, 2005).

### Experimental

A mixture of *p*-Chlorophenylhydrazing hydrochloride (0.02 mol) and methyl acrylate (0.02 mol) was stirred in ethanol (30 ml) at 353 K for 2 h to afford the title compound (yield 50%).  Colourless bars of (I) were obtained by recrystallization from acetone at room temperature.

### Refinement

The O-bound H atom was located in a difference map and freely refined. The C-bound H atoms were positioned geometrically and allowed to ride on their parent atoms, with C—H = 0.93–0.96 Å and with *U*_iso_(H) = 1.2*U*_eq_ of the parent atoms.

### Crystal data

**Table:**

C_9_H_7_ClN_2_O	*F*(000) = 400
*M**_r_* = 194.62	*D*_x_ = 1.494 Mg m^−^^3^
Monoclinic, *P*2_1_/*c*	Mo *K*α radiation, λ = 0.71073 Å
Hall symbol: -P 2ybc	Cell parameters from 1171 reflections
*a* = 9.6461 (19) Å	θ = 3.5–27.5°
*b* = 13.833 (3) Å	µ = 0.40 mm^−^^1^
*c* = 6.5045 (13) Å	*T* = 293 K
β = 94.33 (3)°	Bar, colourless
*V* = 865.4 (3) Å^3^	0.11 × 0.09 × 0.08 mm
*Z* = 4	

### Data collection

**Table:**

Bruker SMART CCD diffractometer	1171 reflections with *I* > 2σ(*I*)
Radiation source: fine-focus sealed tube	*R*_int_ = 0.038
graphite	θ_max_ = 24.5°, θ_min_ = 3.5°
ω scans	*h* = −11→11
5771 measured reflections	*k* = −16→16
1357 independent reflections	*l* = −7→7

### Refinement

**Table:**

Refinement on *F*^2^	Secondary atom site location: difference Fourier map
Least-squares matrix: full	Hydrogen site location: inferred from neighbouring sites
*R*[*F*^2^ > 2σ(*F*^2^)] = 0.059	H atoms treated by a mixture of independent and constrained refinement
*wR*(*F*^2^) = 0.146	*w* = 1/[σ^2^(*F*_o_^2^) + (0.0166*P*)^2^ + 1.7755*P*] where *P* = (*F*_o_^2^ + 2*F*_c_^2^)/3
*S* = 1.24	(Δ/σ)_max_ < 0.001
1357 reflections	Δρ_max_ = 0.23 e Å^−^^3^
122 parameters	Δρ_min_ = −0.32 e Å^−^^3^
0 restraints	Extinction correction: *SHELXL97* (Sheldrick, 2008), Fc^*^=kFc[1+0.001xFc^2^λ^3^/sin(2θ)]^-1/4^
Primary atom site location: structure-invariant direct methods	Extinction coefficient: 0.049 (5)

### Special details

**Table:**

Geometry. All e.s.d.'s (except the e.s.d. in the dihedral angle between two l.s. planes) are estimated using the full covariance matrix. The cell e.s.d.'s are taken into account individually in the estimation of e.s.d.'s in distances, angles and torsion angles; correlations between e.s.d.'s in cell parameters are only used when they are defined by crystal symmetry. An approximate (isotropic) treatment of cell e.s.d.'s is used for estimating e.s.d.'s involving l.s. planes.
Refinement. Refinement of *F*^2^ against ALL reflections. The weighted *R*-factor *wR* and goodness of fit *S* are based on *F*^2^, conventional *R*-factors *R* are based on *F*, with *F* set to zero for negative *F*^2^. The threshold expression of *F*^2^ > σ(*F*^2^) is used only for calculating *R*-factors(gt) *etc*. and is not relevant to the choice of reflections for refinement. *R*-factors based on *F*^2^ are statistically about twice as large as those based on *F*, and *R*- factors based on ALL data will be even larger.

### Fractional atomic coordinates and isotropic or equivalent isotropic displacement parameters (Å^2^)

**Table:**

	*x*	*y*	*z*	*U*_iso_*/*U*_eq_	
Cl1	1.03021 (14)	0.13637 (11)	−0.3127 (2)	0.0771 (6)	
O1	0.3291 (3)	0.0636 (2)	0.5246 (5)	0.0508 (8)	
H1	0.377 (5)	0.019 (3)	0.588 (7)	0.061*	
N1	0.5047 (3)	0.1156 (2)	0.1005 (5)	0.0361 (8)	
N2	0.5009 (3)	0.0759 (2)	0.2928 (5)	0.0380 (8)	
C1	0.3807 (4)	0.1554 (3)	0.0396 (7)	0.0458 (10)	
H1A	0.3588	0.1868	−0.0851	0.055*	
C2	0.2936 (4)	0.1420 (3)	0.1900 (6)	0.0443 (10)	
H2B	0.2015	0.1617	0.1908	0.053*	
C3	0.3726 (4)	0.0919 (3)	0.3442 (6)	0.0386 (9)	
C4	0.6308 (4)	0.1525 (3)	−0.1997 (6)	0.0423 (10)	
H4A	0.5476	0.1707	−0.2710	0.051*	
C5	0.7541 (5)	0.1583 (3)	−0.2943 (6)	0.0467 (11)	
H5A	0.7539	0.1807	−0.4292	0.056*	
C6	0.8760 (5)	0.1314 (3)	−0.1905 (7)	0.0475 (11)	
C7	0.8777 (4)	0.0988 (3)	0.0093 (7)	0.0489 (11)	
H7A	0.9613	0.0808	0.0792	0.059*	
C8	0.7556 (4)	0.0929 (3)	0.1066 (6)	0.0414 (10)	
H8A	0.7570	0.0710	0.2419	0.050*	
C9	0.6311 (4)	0.1195 (2)	0.0022 (6)	0.0339 (9)	

### Atomic displacement parameters (Å^2^)

**Table:**

	*U*^11^	*U*^22^	*U*^33^	*U*^12^	*U*^13^	*U*^23^
Cl1	0.0584 (9)	0.1009 (11)	0.0757 (10)	−0.0004 (7)	0.0298 (7)	0.0190 (7)
O1	0.0422 (17)	0.065 (2)	0.0469 (18)	0.0123 (14)	0.0141 (15)	0.0138 (14)
N1	0.0355 (18)	0.0400 (17)	0.0322 (17)	0.0012 (13)	−0.0011 (15)	0.0040 (13)
N2	0.0361 (19)	0.0440 (18)	0.0338 (18)	0.0036 (13)	0.0011 (15)	0.0065 (14)
C1	0.044 (2)	0.051 (2)	0.040 (2)	0.0064 (18)	−0.009 (2)	0.0060 (18)
C2	0.034 (2)	0.051 (2)	0.048 (3)	0.0073 (17)	0.000 (2)	−0.0012 (18)
C3	0.036 (2)	0.038 (2)	0.042 (2)	0.0023 (16)	0.0044 (19)	−0.0017 (16)
C4	0.045 (2)	0.045 (2)	0.035 (2)	−0.0016 (17)	−0.007 (2)	0.0043 (16)
C5	0.058 (3)	0.048 (2)	0.035 (2)	−0.0062 (19)	0.007 (2)	0.0046 (17)
C6	0.048 (3)	0.045 (2)	0.052 (3)	−0.0038 (18)	0.015 (2)	0.0037 (19)
C7	0.039 (2)	0.052 (2)	0.055 (3)	0.0012 (18)	0.004 (2)	0.008 (2)
C8	0.042 (2)	0.047 (2)	0.035 (2)	0.0014 (17)	0.0012 (19)	0.0063 (17)
C9	0.039 (2)	0.0323 (19)	0.030 (2)	−0.0027 (15)	−0.0007 (17)	−0.0013 (14)

### Geometric parameters (Å, °)

**Table:**

Cl1—C6	1.740 (4)	C4—C5	1.382 (6)
O1—C3	1.334 (5)	C4—C9	1.390 (5)
O1—H1	0.86 (5)	C4—H4A	0.9300
N1—C1	1.349 (5)	C5—C6	1.363 (6)
N1—N2	1.369 (4)	C5—H5A	0.9300
N1—C9	1.420 (5)	C6—C7	1.374 (6)
N2—C3	1.325 (5)	C7—C8	1.381 (6)
C1—C2	1.349 (6)	C7—H7A	0.9300
C1—H1A	0.9300	C8—C9	1.384 (5)
C2—C3	1.396 (5)	C8—H8A	0.9300
C2—H2B	0.9300		
			
C3—O1—H1	116 (3)	C9—C4—H4A	120.0
C1—N1—N2	110.4 (3)	C6—C5—C4	120.2 (4)
C1—N1—C9	128.7 (3)	C6—C5—H5A	119.9
N2—N1—C9	120.6 (3)	C4—C5—H5A	119.9
C3—N2—N1	104.7 (3)	C5—C6—C7	120.4 (4)
N1—C1—C2	108.5 (4)	C5—C6—Cl1	119.9 (3)
N1—C1—H1A	125.7	C7—C6—Cl1	119.7 (4)
C2—C1—H1A	125.7	C6—C7—C8	120.2 (4)
C1—C2—C3	104.7 (4)	C6—C7—H7A	119.9
C1—C2—H2B	127.7	C8—C7—H7A	119.9
C3—C2—H2B	127.7	C7—C8—C9	119.9 (4)
N2—C3—O1	122.3 (3)	C7—C8—H8A	120.1
N2—C3—C2	111.8 (3)	C9—C8—H8A	120.1
O1—C3—C2	125.9 (4)	C8—C9—C4	119.4 (4)
C5—C4—C9	119.9 (4)	C8—C9—N1	120.8 (3)
C5—C4—H4A	120.0	C4—C9—N1	119.8 (3)
			
C1—N1—N2—C3	0.5 (4)	C5—C6—C7—C8	0.2 (6)
C9—N1—N2—C3	174.5 (3)	Cl1—C6—C7—C8	−178.8 (3)
N2—N1—C1—C2	−0.3 (4)	C6—C7—C8—C9	0.2 (6)
C9—N1—C1—C2	−173.7 (3)	C7—C8—C9—C4	−0.3 (6)
N1—C1—C2—C3	−0.1 (5)	C7—C8—C9—N1	−178.9 (3)
N1—N2—C3—O1	−179.6 (3)	C5—C4—C9—C8	0.1 (6)
N1—N2—C3—C2	−0.6 (4)	C5—C4—C9—N1	178.6 (3)
C1—C2—C3—N2	0.4 (5)	C1—N1—C9—C8	165.9 (4)
C1—C2—C3—O1	179.4 (4)	N2—N1—C9—C8	−6.9 (5)
C9—C4—C5—C6	0.3 (6)	C1—N1—C9—C4	−12.7 (6)
C4—C5—C6—C7	−0.4 (6)	N2—N1—C9—C4	174.5 (3)
C4—C5—C6—Cl1	178.6 (3)		

### Hydrogen-bond geometry (Å, °)

**Table:**

*D*—H···*A*	*D*—H	H···*A*	*D*···*A*	*D*—H···*A*
O1—H1···N2^i^	0.86 (4)	1.89 (4)	2.744 (4)	173 (4)

Symmetry codes: (i) −*x*+1, −*y*, −*z*+1.
